# Left extrastriate body area is sensitive to the meaning of symbolic gesture: evidence from fMRI repetition suppression

**DOI:** 10.1038/srep31064

**Published:** 2016-08-16

**Authors:** Agnieszka Kubiak, Gregory Króliczak

**Affiliations:** 1 Action and Cognition Laboratory, Institute of Psychology, Department of Social Sciences, Adam Mickiewicz University in Poznań, Poznań, Poland

## Abstract

Functional magnetic resonance imaging (fMRI) adaptation (a.k.a. repetition suppression) paradigm was used to test if semantic information contained in object-related (transitive) pantomimes and communicative (intransitive) gestures is represented differently in the occipito-temporal cortex. Participants watched 2.75 s back-to-back videos where the meaning of gesture was either repeated or changed. The just observed (typically second) gesture was then imitated. To maintain participants’ attention, some trials contained a single video. fMRI adaptation –signal decreases– for watching both movement categories were observed particularly in the lateral occipital cortex, including the extrastriate body area (EBA). Yet, intransitive (vs. transitive) gesture specific repetition suppression was found mainly in the left rostral EBA and caudal middle temporal gyrus- the rEBA/cMTG complex. Repetition enhancement (signal increase) was revealed in the precuneus. While the whole brain and region-of-interest analyses indicate that the precuneus is involved only in visuospatial action processing for later imitation, the common EBA repetition suppression discloses sensitivity to the meaning of symbolic gesture, namely the “*semantic what”* of actions. Moreover, the rEBA/cMTG suppression reveals greater selectivity for conventionalized communicative gesture. Thus, fMRI adaptation shows higher-order functions of EBA, its role in the semantic network, and indicates that its functional repertoire is wider than previously thought.

Recent behavioral, neuroimaging, and neuropsychological evidence^1^,^2^,^3^,^4^ indicates that performance of meaningful hand movements typically engages a common left-lateralized praxis representation network (PRN)^2^, regardless of whether these are object- (e.g., tool use/*transitive*) or non-object-related (*intransitive*) gestures. Moreover, there is now convincing evidence that the latter category of skilled movements (also referred to as communicative gestures) invokes these same neural resources less than pantomimed tool use^1^,^2^. These conclusions are, nevertheless, based almost entirely on research involving simulated actions retrieved from stored representations^2^,^5^ or gesture imitation^4^,^6^,^7^. Relatively little is known on whether or not a similar common network would also underlie discrimination or recognition of both gesture categories, especially when their processing precedes imitation of the observed movements^8^,^9^,^10^. Even if a common network is engaged in watching transitive and intransitive gesture but, besides differences in the strength of its engagement, there are locally distinct mechanisms devoted to certain aspects of stimulus processing^11^,^12^, it should be possible to identify these disparate functional subdivisions utilizing an fMRI adaptation paradigm^13^. An example of an adaptation trial used in this study, with its structure and timing is shown in Fig. 1.

fMRI adaptation capitalizes on the repetition suppression (RS) effects^14^ linked to stimulus-specific decreases in neural activity, as measured by the blood oxygen level dependent (BOLD) signal. RS is typically observed for back-to-back stimuli^15^, including their characteristic features, and repeated actions directed towards them^16^, but was also demonstrated for performed back-to-back meaningful gestures^17^. The mechanisms that may underlie the observed fMRI adaptation effects include the sharpening of the neural response^18^, its facilitation/accumulation^19^, or even neural fatigue^20^. Regardless of the mechanism(s) involved, the paradigms utilizing RS seem to be most effective to investigate specific neural responses which cannot be distinguished with traditional event-related fMRI paradigms or, even the more so, with block designs.

We expected that the patterns of neural adaptation within a common network depends on a contribution of a given area to visual processing of transitive and/or intransitive gestures. The emphasis on encoding basic features of movement kinematics would lead to a different neural response from encoding of movement goals, including the meaning of the performed action^16^,^17^,^21^. Thus, in the former – linked to lower-level processing – adaptation (if any) could be simply due to fatigue. Conversely, in the higher-order areas any adaptation would result from genuine sensitivity to action meaning. Of course, this same area can show differential adaptation patterns for different gesture categories for both of the reasons. Specifically, the more complex nature of transitive gestures may drive an area to a greater extent and, at the same time, result in less (or little) adaptation due to longer time required to retrieve/assemble information related to its meaning. Because the meaning of intransitive gestures is given in its full form in hand posture and/or accompanying movements, the retrieval of the associated *semantics* should be easier and promptly lead to robust adaptation.

Consistent with earlier reports on meaningful manual actions, and their links to language representations^2^,^9^,^22^, fMRI adaptation for both categories of the symbolic (transitive and intransitive) gestures studied here was found within a common network, including the left extrastriate body area (EBA). Interestingly, its rostral subdivision (rEBA), and the nearby section of the left caudal middle temporal gyrus (cMTG) showed greater sensitivity to visual processing of intransitive gestures. This area – the rEBA/cMTG complex – revealing selectivity for intransitive actions was not detected before with traditional fMRI approaches^2^,^3^. Interestingly, although imitation of the just seen hand movements was also primarily mediated by a common network (with its sensorimotor subdivisions engaged more by transitive pantomimes), a contrast of intransitive vs. transitive gesture imitation revealed a cluster of significant signal modulations in the medial prefrontal cortex (i.e., outside of the common network). It is of note that further analyses revealed that this region shows weaker neural inhibition during imitation of intransitive gestures. This latter outcome is more consistent with a long standing idea that performance of transitive and intransitive gestures requires partly dissociable neural substrates^4^,^23^,^24^.

## Results

### Gesture watching

Watching back-to-back transitive and intransitive gestures, regardless of whether first or second, was associated with virtually the same neural activity when compared to the resting baseline. Therefore, Fig. 2A shows all the areas that were engaged for processing both of the studied gesture categories. In addition to the bilateral involvement of the lateral occipital (LO) cortex, significant signal increases extended ventrally to the to the fusiform gyri (FusG), and antero-dorsally, via the extrastriate body area (EBA), to the posterior divisions of the superior temporal sulci (STS). The dorsal extrastriate activity in both hemispheres was projected to the intraparietal sulci (IPS), including their caudal, middle and anterior divisions, and their immediate vicinities in the superior parietal lobules (SPL). On the medial surfaces the activity in the calcarine sulci (CalcS) extended ventrally to the lingual (Ling) gyri and anteriorly via the parahippocampal gyrus to the hippocampus on the right, whereas dorsally this activity spread to the parieto-occipital (PO) sulci. In the frontal cortex, significant increases were found bilaterally in the mid precentral gyrus, i.e. superior divisions of the ventral premotor cortex (PMv). There was also a small cluster of common engagement in the medial prefrontal cortex on the right.

Nevertheless, despite these commonalities, a direct contrast of all transitive *versus* intransitive gestures (regardless of whether first or second) revealed several regions with significantly greater neural activity, primarily in the left hemisphere. As shown in Fig. 2B there was a large cluster of increased signal in the occipito-temporal cortex, including the caudal middle temporal gyrus (cMTG) and, more dorsally, the rostral divisions of the extrastriate body area (rEBA). In the parietal lobe, in addition to the anterior supramarginal gyrus (SMG), the rostral SPL activity extended via the sensorimotor regions to the dorsal premotor cortex (PMd), and medially to the anterodorsal precuneus (adPreCun). More inferiorly on the medial surface, the mid PO significant signal increase extended into ventrocaudal precuneus (vcPreCun). There was also some right hemisphere involvement of adPreCun. The inverse contrast comparing watching all intransitive *versus* transitive gestures did not reveal any significant increases of brain activity.

### Repetition suppression effects for gesture watching

When neural activity related to watching the same second vs. first transitive and/or intransitive gesture was compared, the most conspicuous clusters of decreased activity were found bilaterally in LO. Smaller clusters of repetition suppression were also found medially in the occipital pole and in the pregenual anterior cingulate cortex (pACC). These effects are shown in Fig. 2C. It is of note that repetition suppression for intransitive gestures was a bit more widespread, particularly in the vicinity of LO.

A direct contrast comparing the neural activity associated with watching the repeated transitive vs. intransitive gesture with the same meaning, i.e. looking for any brain areas that show stronger signal adaptation for intransitive gestures (and as typically expected, revealing repetition suppression), disclosed signal differences in the left rEBA/cMTG complex, as well as in the left vcPreCun and adPreCun. These outcomes are shown in Fig. 2D. A very small cluster of adaptation was also detected in the right EBA. The inverse contrast looking for stronger signal adaptation following repetition of transitive (vs. intransitive) gestures was empty.

Peak coordinates for all the major clusters or their local maxima from the main contrasts of this study, as well as from the localizer scans, are listed in Table 1A–N in Supplemental materials.

#### ROI analyses

The goal of the major ROI analyses was to test for the exact patterns of signal modulations within the critical functional areas or their subdivisions identified independently in our localizer scans, and in the previous studies on the two gesture categories and/or action guidance, namely EBA, cMTG^2^, and dorsocaudal precuneus (dcPreCun)^25^, as well as in the human visual motion selective area hMT+^26^. Supplemental ROI analyses were also performed in the independently defined subdivisions of the clusters revealed by the critical contrast comparing adaptation to the same intransitive and transitive gestures. (These ROIs were rEBA, cMTG; adPreCun, and vcPreCun.).

In the left lateral occipito-temporal ROIs (see Fig. 3A), a 2 (ROI: EBA, cMTG) by 2 (gesture: transitive, intransitive) by 3 (context: first gesture, second same, second different) repeated-measures ANOVA revealed a main effect of *gesture* (F_(1,11)_ = 6.366; p < 0.05; _p_η^2^ = 0.367; alpha = 0.633), such that watching transitive gestures was associated with significantly higher signal increases as compared to watching intransitive gestures. There was also a significant *ROI by context* interaction (F_(2,22)_ = 6.858; p < 0.01; _p_η^2^ = 0.384; alpha = 0.881), such that only for the EBA ROI and the repeated symbolic gestures with the same meaning there was a significant signal decrease. No other interaction or a main effect of context was significant.

Given that in the whole brain analysis significant category specific adaptation effects were observed only for watching intransitive gestures, and in the lateral occipito-temporal ROIs a significant ROI by context interaction was primarily driven by signal modulations in EBA, the brain responses to intransitive gestures across the three contexts were further scrutinized in the EBA ROI. As can be seen in Fig. 3B (in the inset on the right) the *a priori* t-tests revealed that not only there was a significant signal decrease following the presentation of intransitive gesture with the same meaning, but this adaptation effect was significantly greater than signal decreases associated with the presentation of the second movie with a different meaning or a category switch. More detailed analyses related to signal changes limited to the lateral occipito-temporal cluster showing greater adaptation for intransitive (vs. transitive) gestures, with its subdivisions demarcated by these same independent localizers, are shown in Supplemental Figure 1A. The relationships of these ROIs (i.e., their independence from or overlap) with other functional areas identified in this vicinity are depicted in Supplemental Figure 1B.

In the dorsocaudal precuneus ROI (as defined by Hutchison and collaborators^25^) there was only a significant main effect of *context* (F_(2,22)_ = 10.995; p < 0.001; _p_η^2^ = 0.500; alpha = 0.981), such that significantly greater signal increases were observed only between responses to the first and the second movies, regardless of whether the meaning was repeated or not (Bonferroni corrected p < 0.05 in both cases). The responses to the second movies did not differ between themselves (p = 1.0). These effects are depicted in Fig. 3C. More detailed analyses related to signal changes observed in the independently-defined subdivisions of the left vcPreCun and adPreCun ROI are shown in Supplemental Figure 1C.

A human homologue of the visual motion-sensitive area, namely *the hMT*+ *ROI*, was of special interest here. Previous research indicates that the reported differences between representations of transitive and intransitive gestures during their perception and/or production might be primarily due to movement complexity^1^,^2^. If this is really the case, then hMT+ should reveal significantly greater signal associated with watching repeated transitive gestures. Counter to this prediction, this is not what we observed. In the hMT+ ROI, as defined independently in our localizer task, there was no main effect of *gesture* (F_(1,11)_ = 2.405; p = 0.149) which indicates that both kinds of gestures engage this area to more or less the same degree. Consistent with this notion is the obtained main effect of *context* (F_(2,22)_ = 17.092; p < 0.001; _p_η^2^ = 0.609; alpha = 0.999), such that regardless of gesture category any repeated presentation of a gesture video (whether the same or different in meaning) resulted in substantial neural adaptation (Bonferroni corrected p < 0.01 in both cases). Importantly, the *gesture* by *context* interaction was not significant (F_(2,22)_ = 0.140; p = 0.870). Such observations are of great importance because, despite any differences in movement complexity in favor of transitive gestures, the relatively lower-level features of the two kinds of movements (i.e., less kinematics required to achieve a meaningful posture for intransitive gestures) do not seem to matter for the area specialized in motion processing. In fact, the unveiled effects indicate that most of the signal decrease observed in hMT+ for back-to-back movies might be due to sparser coding required to process the similarly structured and repeated patterns of motion, and/or possibly some neural fatigue. Therefore, these effects will not be discussed any further.

### Gesture imitation

During imitation of the just-seen manual actions, both transitive and intransitive gestures (when compared to resting baseline) engaged nearly the same areas. Therefore, Fig. 4A shows all the areas that were engaged for imitation of both of the studied gesture categories. There were significant signal increases spanning the rostral SPL, via the sensorimotor cortices, and precentral sulcus. The PMv, as well as the nearby opercular and insular cortices were engaged bilaterally. On the medial surfaces, there was a large cluster of increased bilateral activity comprising the supplementary and pre-supplementary motor area (SMA complex) and cingulate motor area (CMA). There was also bilateral signal increase in the putamen, and the left thalamus.

Although virtually the same functional regions mediated their imitation, a direct contrast of transitive *versus* intransitive gestures nevertheless revealed greater engagement of left SMA and PMd, with some activity extending via the sensorimotor cortices to superior divisions of rostral SPL. The inverse contrast (imitation of intransitive vs. transitive gestures) revealed greater activity in the right insular cortex extending onto PMv, and a cluster in the dorsomedial prefrontal cortex (DMPC). These effects are shown in Fig. 4B. As corroborated by further ROI analyses performed within the confines of the paracingulate gyri, DMPC showed significantly weaker inhibition of activity for intransitive gestures. The ROI, the investigated cluster, and the obtained effect is depicted in Supplemental Figure 2.

A schematic comparison of neural activity related to gesture imitation and gesture watching is shown in Fig. 5A.

### “Same-different” discrimination test.

Repeated-measures ANOVA on mean reaction times (RTs) for the follow-up same-different discrimination test showed a significant main effect of *gesture* (F_(1,10)_ = 45.5, p < 0.001, _p_η^2^ = 0.82, alpha = 1) indicating facilitated processing of intransitive category regardless of the study context (i.e., whether the meaning stayed the same or changed). The effect of *context* was also significant (F_(2,20)_ = 7.02, p < 0.05, _p_η^2^ = 0.413, alpha = 0.884) and was such that discrimination of gestures with between-category changes in meaning was easier compared to both gestures with the same meaning (Bonferroni corrected p < 0.05) or even different meaning, but still belonging to a given category (Bonferroni corrected p < 0.01). The *gesture* by *context* interaction, however, was not significant (F_(2,20_ = 1.14; p = 0.340). The mean response times for all the conditions are shown in Fig. 5B.

## Discussion

Consistent with recent behavioral and neuroimaging results^1^,^2^, we find evidence that both categories of the symbolic gestures that we studied, that is tool use (transitive) pantomimes and communicative (intransitive) gestures, share common representational resources. This is also evident in the extrastriate body area – a functional region never considered in the context of symbolic manual actions – which as a whole shows similar sensitivity to both gesture categories. If any differential processing between the two categories is observed, it can be linked primarily to differences in their complexity, perhaps including greater kinematic demands but also, and arguably more importantly, the necessity for retrieval of a related object (or at least its function) for transitive actions^2^. In agreement with earlier research, our study also shows that the areas most critical for gesture representations are left lateralized. That is, although the neural activity associated with visual processing of the studied gestures was largely bilateral, the direct contrasts of the two categories revealed modulations primarily in the left hemisphere^2^,^10^. Greater signal increases specific to watching transitive gestures were found in the left occipito-temporal cortex and in the left posterior parietal lobe (aSMG, adPreCun, and vcPreCun). Moreover, similarly to earlier reports^2^,^10^, we also observed greater left-lateralized sensorimotor involvement extending substantially to the dorsal premotor cortex.

Most importantly, consistent with an earlier study on specificity of action processing^27^, adaptation effects for repeated gestures were found bilaterally and were located in the occipital, parietal, as well as in the medial prefrontal regions. Yet, greater repetition suppression for watching intransitive gestures was exclusively left lateralized and observed only in the occipito-temporal cortex, a region critical for storing concepts related to both categories of actions^2^,^3^. Interestingly, the signal decreases were found in the vicinity that, regardless of the testing condition, in the current study was typically more active for transitive gestures, and were limited to rEBA and cMTG. Our results indicate that the rEBA/cMTG cluster is either a single higher-order functional area for action observation or rEBA transforms the cMTG inputs (putatively representing the concepts of actions) into higher-order symbolic representations. Finally, although this same contrast (showing greater adaptation for intransitive gestures) revealed two additional clusters in the precuneus, the more careful examination of the signal change patterns unveiled repetition enhancement effects. Although these signal increases were greater for transitive gestures in the context of repetition of the same meaning, they were, nevertheless, similar across both contexts for both categories of the symbolic gestures that we studied.

All in all, these results are in agreement with the notion that the areas most critical for processing tool use pantomimes and familiar intransitive gestures are located in the left-lateralized praxis representation network^2^,^22^,^28^. More importantly, this study indicates that – within the occipito-temporal cortex – not only cMTG^29^ but also EBA contributes significantly to the processing of action concepts or even their semantic content. Indeed, in contrast to earlier proposals^30^,^31^, arguably a single functional area, containing only rostral divisions of EBA and a small subdivision of cMTG – that is rEBA/cMTG complex – shows greater sensitivity to the sequences of movements and/or meaning of intransitive gestures. In the the precuneus, conversely, the build-up of activity (particularly striking for transitive gestures when their meaning is repeated) is consistent with the notion that this region is more responsive to the observed and/or planned visuospatial hand movement kinematics, rather than action goals or their meaning.

### Adaptation patterns consistent with action concept processing in the left caudal middle temporal gyrus

Although focal lesions involving cMTG also lead to semantic or language comprehension deficits^32^, the left cMTG is frequently linked to conceptual representations of common manipulable objects, the associated actions, and/or the visual processing of tool-related features^2^,^3^,^33^,^34^,^35^,^36^,^37^,^38^. Because this area and the nearby regions are strongly connected with SMG^39^,^40^,^41^,^42^,^43^, it is not surprising that neuropsychological evidence shows that damage in this vicinity often results in impaired performance on manual tasks requiring access to such concepts/knowledge^44^. Our study shows that a few subdivisions of a larger area often referred to as cMTG are clearly involved in the processing of both categories of the symbolic gestures. Yet as a whole, cMTG does not show any differential or even any particular sensitivity to gesture during the watching task. Only its subdivision somewhat overlapping with the area for hand-independent praxis planning^3^, with its inferior part not even activated for gesture watching above the resting baseline, showed sensitivity (signal decreases) to intransitive gestures. Namely, there was little decrease of activity following presentation of the repeated transitive gestures, whereas adaptation to repeated intransitive gestures was clearly present. Importantly, although the whole brain analyses indicated that this vicinity was in fact more engaged in watching transitive (tool use) gestures, the ROI analysis limited only to its subdivision that was common with cMTG as identified in our earlier study^2^ showed that its initial response to both gesture categories was similar. Thus, neither the less complex movement kinematics nor the absence of an object (for the gesture to be understood) can explain the presence of adaptation for one gesture category. The observed effect, therefore, indicates a particular sensitivity of cMTG to the sequences of *communicative* hand movements or actions leading to meaningful postures that do not involve objects. It is worth emphasizing again, though, that only a small fraction of what is typically referred to as cMTG^2^,^3^ shows this specific pattern of activity, and the inferred sensitivity. Even more importantly, it seems to do it in concert with rEBA.

### Adaptation to action meaning (semantics) in the left extrastriate body area

Although the area specialized for processing/representing body parts was identified at the turn of the century^45^, this functional region – dubbed the extrastriate body area – has lately received a lot of attention. It is typically located near posterior divisions of the inferior temporal sulcus (pITS) and cMTG^31^,^46^ but there is also evidence pointing to its more superior location within the occipital lobe – in the proximity of the superior temporal sulcus (STS)^30^,^47^, right above the temporo-occipital region sensitive to haptic skills^26^. Neuroimaging investigations also find that in right-handed individuals EBA might be greater on the right^48^. It has been shown to be engaged not only in processing of body parts, including hands, but also in the control of the moving hands and mental motor imagery^47^. Notably, there is already some evidence that EBA might be involved in representing knowledge of action concepts^27^. In this specific sense, EBA would be decoupled from the motor systems^49^ (although there are also results suggesting close links^50^).

Despite quite recent evidence that EBA is not engaged in higher-order functions^51^, our results clearly indicate that this area shows robust tuning of processing devoted to symbolic gestures. The putative sharpening of its responses cannot be limited to action meaning, though, because a similar trend towards signal decrease is also observed for gestures with a different meaning (pointing to partial sensitivity to relatively lower-level features of these symbolic actions). Only the rostral division of EBA, as shown by the whole brain contrast of the two gesture categories during watching the clips with same meaning, or the whole EBA as shown by a priori tests, shows specific sensitivity to the meaning of intransitive gestures. There are at least two sources of evidence supporting such a conclusion. There was substantially smaller decrease of activity in EBA for back-to-back transitive gestures while adaptation associated with repetition of intransitive gestures was robust. Yet, because there was also a significant signal difference for watching the first movies, the greater decrease of activity would be less compelling were it not for the fact that only for intransitive gestures the adaptation to meaning (the same gesture from a different view) was significantly greater than the one observed for any other repeated (putatively lower-level) features contained in the second movie with disparate meaning. In sum, the lack of adaptation for repeated tool-use pantomimes is taken here as evidence that rEBA is either excessively engaged in processing of low-level movement features or incapable of managing the semantic content of tool use gestures (or both). This might be, for example, also due to an inability to retrieve a relevant object that would complete the meaning of the observed action. In sharp contrast, the ease of initial processing and dramatic decreases of activity associated with repeated intransitive gestures, again, point to exceptional and inherent responsiveness of rEBA to the meaning of the observed symbolic movements that can be expressed only with the hand or are deeply conventionalized.

Counter to reports suggesting that EBA, although putatively belonging to a multimodal system that represents action meaning, is not itself capable of semantic processing^30^,^51^, the adaptation effects observed here indicate robust sensitivity to action semantics. Namely, EBA as a whole, as opposed to the whole cMTG, reveals such sensitivity, although it also shows some adaptation following the repeated lower-level features of symbolic gestures. rEBA seems to be even more sensitive to action semantics when the gesture is conventionalized and, therefore, its meaning given directly by the hand. Neither any area in more anterior divisions of the temporal cortex nor in the prefrontal cortices showed adaptation specific to any of the two gesture categories. In contrast, the activity in the precuneus revealed repetition enhancement effects (in one context also greater for transitive gestures) which are here quite likely indicative of activity build-up that must precede the performance/imitation of spatially guided movements^52^.

Of course, as our detailed region-of-interest analyses show, EBA is not a homogenous area and does not show sensitivity to the meaning of symbolic gesture in isolation. Indeed, using independent criteria for delineating functional regions, we see clear evidence for the close collaboration of rEBA and a small subdivision of cMTG (perhaps with rEBA deriving some its functional properties from cMTG). If this is the case, then consistent with earlier accounts of cMTG discussed above, cMTG would be responsible for providing the information on the “conceptual how” for further refinement in rEBA. However, the apparent rEBA/cMTG complex may simply be a single functional area specialized for symbolic hand actions. After all, despite different initial engagement in gesture watching, these subdivisions show strikingly similar patterns of signal modulations (see Supplemental Figure 1). Given all the evidence we have, we postulate that the primary function of the rEBA/cMTG complex is representation of the conventionalized symbolic meaning, rather than the concept of body-part movements required to express such a meaning^53^,^54^. If this was really so then the rEBA could have shown quite similar pattern of signal adaptation for transitive actions with the actual tool in hand.

Behavioral and neuroimaging evidence converge on the interpretation that performance and/or recognition of transitive gestures is more demanding^1^,^2^,^10^,^11^. Our behavioral data are consistent with this notion and clearly indicate that it is much harder to say that the same transitive gesture is shown, regardless of whether it follows the same or different exemplar from the transitive category. Intransitive gestures, on the other hand, are always easier to recognize, irrespective of the context.

### Gesture watching versus imitation

Consistent with recent speculations^27^, but counter to “embodied” views postulating that some aspects of action recognition and performance are mediated by the same systems (e.g. parietal and frontal mirror neurons^55^), none of the areas engaged during gesture watching was then invoked during gesture imitation. This is also the case in the ventral premotor cortex where non-overlapping clusters were recruited for the two tasks. Indeed, a common, predominantly left-lateralized network of areas also mediated the actual imitation of both gesture categories. Notably, as before^2^,^10^, performance of transitive gestures relied on the left sensorimotor and dorsal premotor cortices substantially more. This effect is most likely due to greater kinematic demands imposed by transitive (tool use) gestures. Imitation of intransitive symbolic gestures, conversely, depends also on modulations within bilateral medial prefrontal cortices (which were not revealed in the contrast with resting baseline). Although this region might be critical for mediating first-person perspective taking^56^, our outcomes show that this role extends to meaningful actions typically performed in social contexts.

## Conclusions

The results obtained in this study clearly indicate that fMRI adaptation paradigms (capitalizing on repetition suppression effects) are a valuable tool for studying higher-order motor cognition. Much more sophisticated and lengthier designs would be required to obtain similar effects with traditional block designs or event-related approaches. Once again fMRI adaptation proved to be well suited for studying neural processing at a finer scale, revealing different patterns of activity within the confines of larger functional areas. Thanks to this approach we were able to demonstrate that left EBA, and in particular its rostral subdivision forming an rEBA/cMTG complex, is an important higher-level functional node that is actually sensitive to the meaning of symbolic gestures, not a lower-level unit providing more basic information to other semantic nodes. All in all, our study shows that the rEBA/cMTG complex, as an area somewhat decoupled from the motor systems, can be a part of the network already representing the *semantic what* of visually processed actions.

## Methods

### Participants

Fifty two (52) healthy adult volunteers contributed to the realization of this project. Twelve of them (5 women), with mean age at the time of scanning (MA) = 25.7 years, SD = 4.8 years, participated in the main study. All participants were right-handed with the mean laterality index from the Edinburgh Handedness Inventory (LI_EHI_) = 87, SD = 16 (where 100 indicates a preference for using the right hand in all everyday activities listed, e.g. writing, using scissors, etc.)^57^. The experiment was conducted at *the Lewis Center for Neuroimaging* (LCNI) at the University of Oregon with the approval of the local Ethics Committee for Research Involving Human Subjects. Additional 40 participants (20 women, MA = 23.6, SD = 2.5 years) were tested in localizer scans. All of them were right-handed (LI_EHI_ = 93.01, SD = 9.6). The Bio-Ethics Committee at Poznan University of Medical Sciences approved the experimental protocols. Informed consent was obtained from all 52 participants. Therefore, testing in all paradigms conformed to the WMA Declaration of Helsinki.

### Stimuli

All the transitive and intransitive gestures included in this study are listed in the Appendix (in Supplemental materials, and the actual clips can be downloaded from the open public repository). To be consistent with earlier reports on research involving both categories of these gestures^2^,^3^,^10^,^11^,^22^ and the neuropsychological studies and models related to their neural representations^4^,^23^,^24^, all the gestures, including tool use pantomimes, were filmed and imitated without objects. To make sure that during testing participants would understand all the gestures and would be also familiar with their imitation, prior to testing there was an extensive training with the stimuli having the same meaning and shot from the two perspectives. The actress was the same but she was dressed differently than in the movies shown in the fMRI session. The video stimuli with the same meaning showed gestures from two different views, and possibly contained slightly different movement kinematics, because the clips were shot from two different perspectives (with a difference of 60° angle) at a different time. This was done on purpose to eliminate as much adaptation to lower-level features of these movies as possible.

### Training phase

All participants were very well acquainted with the stimuli and the tasks. First the experimenter briefly described and showed each gesture once, and then there was a 9-minute long practice, wherein participants watched 2.75-s videos with an actress executing gestures with her right hand, and then they imitated the just-seen 24 transitive (tool use) pantomimes, and 24 intransitive (conventionalized) gestures. That is, each of the 12 gestures from both categories was shown and then imitated (by the participant) two times in a randomized order. Each trial was preceded by a centrally displayed 1.5-s gesture name (in a form of gerundive verb-derived noun). All participants were asked if additional training was required. Because the study was a part of a greater project, the videos and the training procedures were similar/identical to the ones used in earlier studies^2^,^3^,^10^,^11^,^22^.

### Experimental design

**Figure 1:**
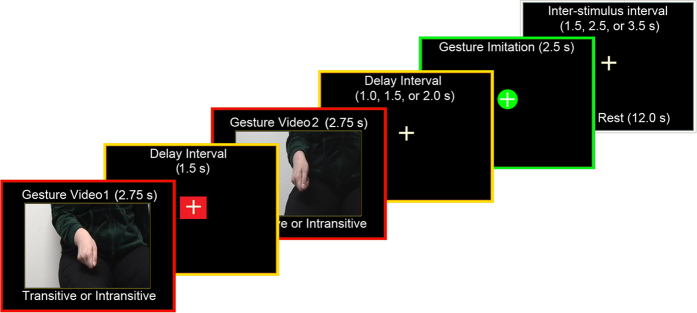
A schematic of the design, trial structure, and timing. A 2.75-s gesture video was followed by a 1.5-s Interstimulus Interval, a second gesture clip of the same duration, and a variable Delay Interval (1.0, 1.5 or 2.0 s). Subsequently, the 2.5-s cue for imitation was shown wherein participants were to imitate the just-seen (typically second) gesture, and a trial concluded with a variable InterTrial Interval of 1.5, 2.5 or 3.5 s. Additional six (12-s) rest intervals were introduced at random in a given run. Only the initial 2.5-s intervals - when the hand was actually moving - were explicitly modeled for gesture watching.

**Figure 2:**
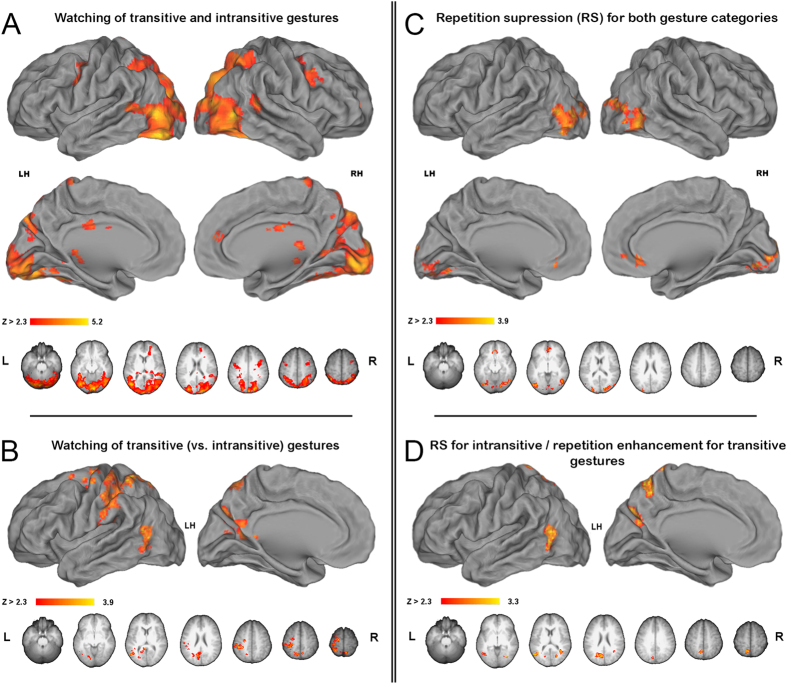
Signal modulations associated with gesture watching. In all figures, group mean statistical parametric maps were obtained using clusters determined by Z > 2.3, and a cluster corrected significance threshold of P = 0.05. Common areas are shown with inclusive contrast masking. (**A**) ***A common network associated with watching transitive and intransitive gestures (vs. baseline)***. The critical clusters on ventro-lateral and dorso-lateral surfaces were found in the fusiform gyrus (FusG), the lateral occipital (LO) cortex, the posterior superior temporal sulcus (STS), the intraparietal sulcus (IPS) and its vicinity in the superior parietal lobule (SPL), and in the mid precentral sulcus, corresponding to the superior division of the ventral premotor cortex (PMv). On the medial surfaces, the clusters were located in the calcarine sulcus (CalcS), the parieto-occipital (PO) sulcus, mid cingulate and anterior cingulate gyrus. (**B**) ***A direct contrast of neural responses for watching all transitive (vs. intransitive) gestures***. The activity was almost exclusive to the left hemisphere, and located in the occipito-temporal cortex, anterior supramarginal gyrus (SMG), rostral SPL, sensorimotor cortex, and dorsal precentral gyrus, corresponding to the dorsal premotor cortex (PMd). On the medial surface the activity was found in the ventrocaudal precuneus (vcPreCun), and anterodorsal precuneus (adPreCun). (**C**) ***Repetition suppression (RS) effects for both gesture categories***. The clusters were located in LO, posterior CalcS/occipital pole, and the pregenual anterior cingulate cortex (pACC). (**D**) ***RS effects exclusive to intransitive gestures***. The clusters were found in the occipito-temporal cortex, vcPreCun, and adPreCun.

**Figure 3:**
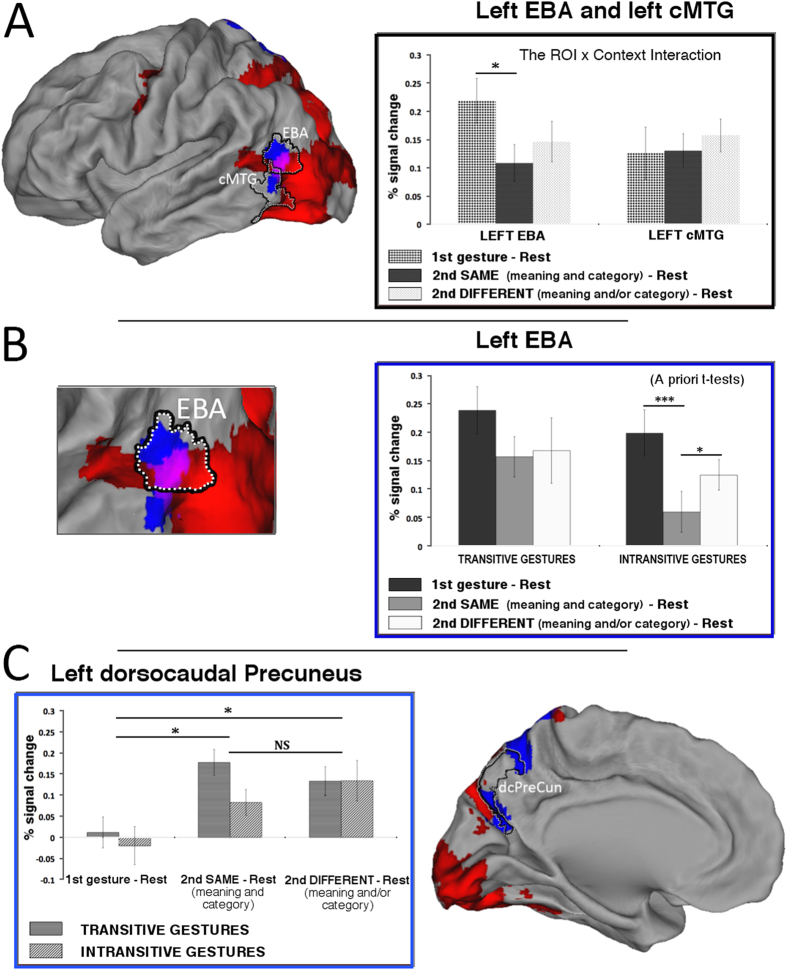
Region of interest (ROI) analyses. (**A**) ***Surface renderings and repetition suppression of the signals in the independently defined extrastriate body area (EBA) and caudal middle temporal gyrus***(***cMTG***). The patterns of activity within the confines of these regions was quite different (as shown by the significant ROI by context interaction). Significant repetition suppression exclusive to the same symbolic gestures was found only in EBA. Although there was also substantial decrease of activity associated with the second video showing a different gesture, it was not significant. (**B**) ***Detailed analyses of the signal decreases associated with watching intransitive gestures.*** As shown by a priori t-tests, the signal decrease associated with watching intransitive gestures with the same meaning was significantly greater from the two other conditions (**C**). ***Repetition enhancement in the dorso-caudal precuneus (dcPreCun), as defined elsewhere***^25^. Significant increases of activity for any of the second movies showing the two gesture categories (i.e., regardless of whether the same or different) were found. Given that the gesture by context interaction was not significant, the substantial difference between activity associated with watching the same transitive and intransitive gestures was not considered here. Red indicates networks of regions with significant signal increases vs. baseline during gesture watching, regardless of the movie context (1^st^, second same, second different). Blue shows areas with stronger adaptation for intransitive gestures outside of these networks. Violet magentas indicate the area where the adaptation for intransitive gestures was found within a network for gesture watching. Except for a priori t-tests, asterisks indicate all the significant differences with the Bonferroni-corrected P values of at least 0.05 (*) or 0.001 (***); ‘NS’ indicates not significant differences.

**Figure 4:**
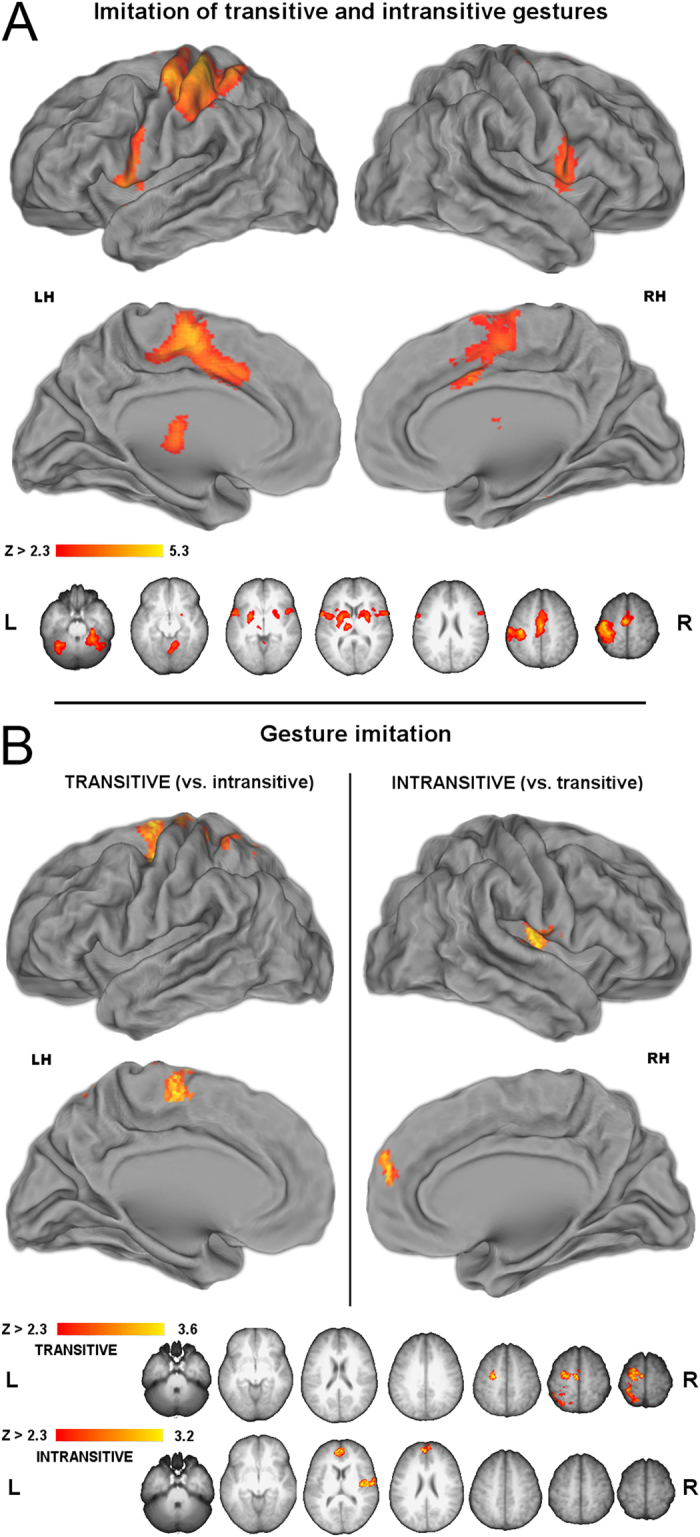
Gesture imitation. (**A**) ***Areas recruited during imitation of both transitive and intransitive gestures***. In the left hemisphere, rostral SPL activity extended via sensimotor cortices to PMd, whereas bilateral activity was observed medially in the SMA complex and cingulate motor area (CMA), and laterally in PMv, and the nearby opercular and insular cortex. (**B**) ***Direct contrasts of neural activity for imitation of transitive vs. intransitive gestures***. (Left panel) Imitation of transitive gestures is linked to significantly greater engagement of left PMd, SMA, but also the sensorimotor cortices and rSPL. (Right panel) Imitation of intransitive gestures is linked to significantly greater engagement of the right paracentral parietal operculum and insula, as well as the bilateral dorsomedial prefrontal cortex (DMPC).

**Figure 5:**
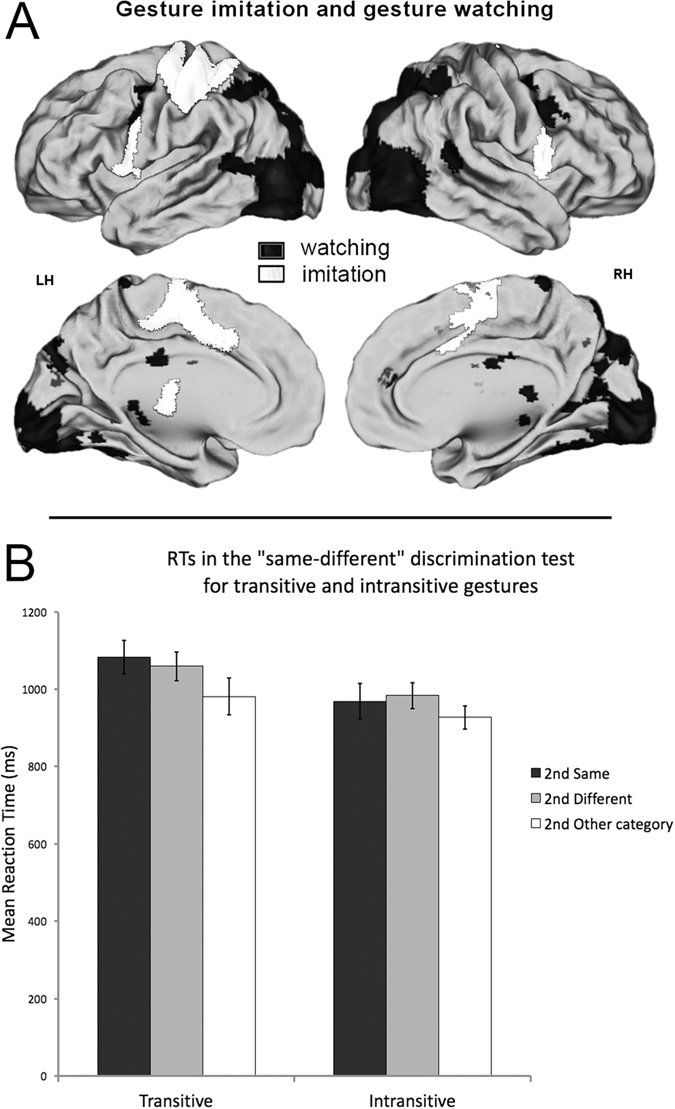
Neural activity and behavioral responses. (**A**) ***Schematic comparison of representations for gesture imitation and gesture watching.*** The critical neural networks are independent for the two tasks. (**B**) ***Reaction times for the ‘same-different’ gesture discrimination.*** Despite common effects of context for both gesture categories, intransitive gestures are discriminated significantly faster, regardless of the context.

In a typical session, there were at least 3 functional runs (12.3 minute long). Participants watched videos showing transitive and/or intransitive gestures and imitated the just-seen, typically second gesture. A single run comprised 24 back-to-back (adaptation) trials, 12 from each gesture category. Trial timing and structure was shown in Fig. 1. To control for attention, additional 24 trials comprised only one gesture to be imitated. In adaptation trials, a third of gestures was repeated with the same meaning but different views and, arguably, somewhat different movement kinematics. The remaining 2/3 of trials contained either a within-category change in meaning, or a change of gesture category. In the majority of participants an additional (8-minute) run was introduced with the emphasis on back-to-back trials with the same meaning. The summary of study conditions is shown in Table 1.

### Stimulus presentation

Gesture videos were displayed using SuperLab 4.0.7b (http://www.superlab.com/) and presented via a back-projection screen visible in a mirror. A central fixation cross restrained eye movements which were continually examined with an MRI-compatible tracking system (http://a-s-l.com). Manual performance was monitored by the experimenter and digitally recorded.

### Data acquisition

Neuroimaging data for the main experiment were acquired using a Siemens (Erlangen, Germany) 3 Tesla Allegra MRI scanner at LCNI. The BOLD echoplanar images were acquired using a T2*-weighted gradient echo sequence: Time Repetition (TR) = 2000 ms; Time to Echo (TE) = 30 ms; Flip Angle (FA) = 80°; 64 × 64 matrix; Field of View (FOV) = 200 mm; 32 contiguous axial-oblique slices with in-plane resolution of 3.1 × 3.1 mm and 4-mm thickness. T1-weighted structural images with 1.0-mm isotropic voxels and 176 contiguous axial slices were acquired with an MP-RAGE pulse sequence: TR = 2000 ms; TE = 4.38 ms; FA = 8.0°; 256 × 176 voxel matrix size; FOV = 256 mm. Localizer data were acquired using Siemens 3T (*Trio* and *Spectra*) scanners at the *Nencki Institute of Experimental Biology* in Warsaw, and *RehaSport Clinic* in Poznan, respectively, using very similar neuroimaging parameters.

Raw DICOM images were converted to FSL NIfTI format with MRIConvert program (http://lcni.uoregon.edu/downloads/mriconvert). Motion correction was typically performed during data acquisition using Siemens EPI-navigated prospective motion correction, followed by an automatic retrospective re-acquisition. FSL 4.1.4^58^ was used for data analyses, and motion correction algorithm (MCFLIRT) was applied if necessary. Prior to statistical analyses the non brain tissue was removed using FSL *Brain Extraction Tool* (BET). Functional data were spatially smoothed using 6.2 mm isotropic Gaussian FWHM kernel. High-pass temporal filtering (σ = 60 s) was estimated directly from the data. For each participant, each functional run was modeled separately at the first level using 10 predictors (in FSL called explanatory variables, EVs). For each gesture category EVs included: first movie, second movie (same meaning, different meaning), their imitation, as well as between category gesture change, and rest. Although clip duration was 2.75 s, only the 2.5-s intervals were modeled when the hand was actually moving. All trials containing one gesture were treated as conditions of no interests. The degrees of freedom were estimated and adjusted for data autocorrelation using the FSL pre-whitening technique. FMRIB’s *Improved Linear Model* (FILM) with local autocorrelation correction was then used for time-series statistical analyses. Registration to individual anatomical and standard space MNI152_T1_2 mm_brain images was carried out with FMRIB’s *Linear Image Registration Tool* (FLIRT). Intersession fixed-effects (single subject, level 2) were tested first, and then intersubject (level 3) random-effects components of mixed-effects variance were modeled and estimated using FMRIB’s *Local Analysis of Mixed Effects* (FLAME) Stage 1 + 2^59^. Z (Gaussianized t/F) statistic images were obtained with cluster sizes determined by FSL’s default Z > 2.3 and cluster corrected significance threshold of P = 0.05.

Region of interests (ROIs) were always defined independently from the main study, using the localizer scans, areas defined in an earlier study^2^, and/or the maps from the Harvard-Oxford probabilistic atlas (implemented in all major neuroimaging programs). The latter approach was also applied to limit the selected activity regions to the confines of these externally defined maps – a procedure already used in an earlier study on these two gesture categories^3^. Similarly, subdivisions of larger areas identified in the main study were always defined independently of this study, again with help from the localizer scans and areas identified before^2^,^3^. FSL Featquery was used to extract mean percent signal change in each condition compared to resting baseline.

Anatomical locations of clusters showing significant signal modulations were determined with help from the *Juelich Histological* and *Harvard-Oxford probabilistic* atlases implemented in FLS, and their functional role was also established with the localizer scans. These clusters were visualized using the CARET software average-fiducial-mapping overlays onto the human population-average, landmark- and surface-based (PALS) brain atlas^60^.

### Localizers

*Extrastriate body area (EBA) localizer* was run two times, typically on two different days. Each run consisted of 4 separate 18-s blocks of greyscale photos of headless bodies (in their underwear) on a white background (19 images of defensive or dance postures, 1 repeated for a one-back task controlling for attention; 6 images of male-female pairs, 13 females only), 4 separate blocks of scenes containing primarily chairs, armchairs, and benches (19 exemplars of such “furniture”, 1 repeated), and 4 separate blocks of their scrambled versions. They were embedded within a larger multilocalizer scan also containing 4 blocks of tools, 4 blocks of non-tool objects, their scrambled counterparts, and 4 rest blocks, all presented in a pseudorandom order. The critical comparison of *bodies vs. furniture* was run first, and the location of activity cluster was further refined by a t-test of *bodies (*–scrambled bodies*) vs. furniture* (*–scrambled furniture*), using a more conservative value of Z > 3.1 and a (corrected) cluster significance threshold of P = 0.05.

#### Human visual motion selective area (hMT+) localizer

It was run two times and, again, typically on two different days. Its structure was the same as the one described before^26^. Two types of radial and concentric gratings were either rotating clockwise and/or counter-clockwise (in 3 different 14-s blocks) or contracting and/or expanding (also in 3 different 14-s blocks), and additional six 14-s blocks with passive viewing of stationary radial (3 blocks), and/or concentric control gratings (3 blocks). They were imbedded within a multi-localizer scan also testing for brain areas sensitive to two kinds of hand movements (with three 14-s blocks of forward and/or backward hand movements, and three 14-s blocks of wrist rotation). With one of the two visual conditions always shown first, they were presented in a pseudorandom order, including additional six (6) 14-s rest periods. The critical comparison of visual responses to *moving vs. stationary patterns* (regardless of their type) was run with a rather conservative approach, i.e., with voxel-corrected threshold value of P = 0.001.

### “Same-different” gesture discrimination

Eleven out of 12 participants from the main fMRI study took part in a follow-up gesture discrimination test. The task was to watch 144 back-to-back gesture clips and decide as quickly and accurately as possible whether the second gesture in each pair was the same or different in its meaning. As before, a 2.75-s gesture video was followed by a short variable delay interval (0.5, 1.0 or 1.5 s) and the subsequent video shot from a different perspective was displayed. Participants were required to press with the left or right index finger, counterbalanced, a left or right key on RB-540 response pad by Cedrus^®^, San Pedro, CA (http://cedrus.com/rb_series/), to indicate whether the gesture was the same or different. Response latency and accuracy were recorded by Dell Latitude D620 PC with SuperLab 4.0.7b. Two repeated-measures Analyses of Variance (ANOVAs) were run, one for discrimination accuracy and one for response times to correctly discriminated gestures. The within-subjects factors were gesture type (transitive, intransitive), and context of repetition (same meaning, different meaning, meaning and category changed). IBM SPSS statistics v. 23 (0.0.2) was used for data analysis and the adopted significance level was ∝ = 0.05.

## Additional Information

**How to cite this article**: Kubiak, A. and Króliczak, G. Left extrastriate body area is sensitive to the meaning of symbolic gesture: evidence from fMRI repetition suppression. *Sci. Rep.* **6**, 31064; doi: 10.1038/srep31064 (2016).

## Supplementary Material

Supplementary Information

## Figures and Tables

**Table 1:** A rapid summary of three possible pairs of back-to-back gestures.

1^st^ video	2^nd^ video
transitive or intransitive gesture	the same gesture (the same meaning but changed view)
transitive or intransitive gesture	a different gesture from the same category
transitive or intransitive gesture	a different gesture from the other category
