# Supplementary material for: Left extrastriate body area is sensitive to the meaning of symbolic gesture: evidence from fMRI repetition suppression
Source: Sci Rep. 2016 Aug 16;6:31064. doi: 10.1038/srep31064 (PMC4985812; doi:10.1038/srep31064)
Supplement: Supplementary Information [file srep31064-s1.pdf]

Left extrastriate body area is sensitive to the meaning of symbolic gesture: evidence from fMRI repetition suppression

Agnieszka Kubiak[1], and Gregory Króliczak[1*]

[1] Action and Cognition Laboratory, Institute of Psychology, Department of Social Sciences, Adam Mickiewicz University in Poznań, Poznań, Poland

*Correspondence: Gregory Króliczak, Laboratorium Badania Działań i Poznania, Instytut Psychologii UAM, ul. Szamarzewskiego 89, 60-568, Poznań, Poland

e-mails: krolgreg@amu.edu.pl; krol.greg@gmail.com

KEYWORDS: transitive, tool use, intransitive, watching, fMRI adaptation, EBA, signal decrease, repetition enhancement

Appendix: Video stimulus lists

Transitive gestures: Cutting, Dialing, Painting, Pounding, Pouring, Reeling, Scrubbing, Sewing, Stirring, Typing, Unlocking, Writing

Intransitive gestures: Beckoning, Counting, Flicking, Hitchhiking, Pointing, Scolding, Shooing, Snapping, Stopping, Talking, Wavering, Waving

Table 1A-N: Peak coordinates for all the major clusters or their local maxima from all the contrasts and localizer scans used in this study

GESTURE WATCHING: MAJOR CONTRASTS FROM THE MAIN EXPERIMENT

A. Watching 1st ***transitive*** *gestures vs. rest*

(Z > 2.3, p = 0.05 cluster corrected)

Region	MNI Coordinates			Peak value
	x	*y*	*z*	z-max
Left Precentral Sulcus	-42	-6	46	4.42
Left Superior Parietal Lobule (SPL)	-36	-56	58	3.36
Left Extrastriate Body Area (EBA) (rostral subdivision)	-50	-66	6	4.87
Left caudal Middle Temporal Gyrus (cMTG) (superior part)	-50	-66	4	3.99
Left MT+	-46	-70	6	4.96
Left Visual cortex V2	-14	-100	12	3.17
Left Lingual Gyrus (LING)	-2	-82	-8	4.04
Left Fusiform Gyrus (FUS/FG)	-16	-88	-16	4.59
Right SPL	28	-60	60	3.81
Right EBA	46	-62	4	4.51
Right MT+	30	-78	-8	4.29
Right Lateral Occipital cortex (LO) (inferior division)	52	-64	-10	4.41
Right Visual cortex V2	16	-94	16	4.23
Right LING	8	-78	-8	4.67
Right FUS/FG	22	-82	-14	4.35

B. Watching 1st ***intransitive*** *gestures vs. rest*

(Z > 2.3, p = 0.05 cluster corrected)

Region	MNI Coordinates			Peak value
	x	*y*	*z*	z-max
Left EBA (rostral subdivision)	-50	-68	8	4.1
Left cMTG (superior division)	-40	-66	4	3.40
Left MT+	-42	-74	6	4.35
Left Visual cortex V2	-14	-102	12	3.26
Left LING	-2	-80	-8	3.91
Left FUS/FG	-36	-58	-16	3.5
Right EBA	46	-62	4	4.68
Right MT+	28	-78	-10	4.56
Right Visual cortex V2	16	-94	16	3.95
Right LING	6	-78	-6	4.65

*C. Watching 2nd **same transitive** gestures vs. rest*

(Z > 2.3, p = 0.05 cluster corrected)

Region	MNI Coordinates			Peak value
	x	*y*	*z*	z-max
Inferior Frontal Gyrus (IFG) pars opercularis	-48	20	4	3.35
Left Middle Frontal Gyrus (MFG)	-40	20	30	3.0
Left Superior Frontal Gyrus (SFG)	-8	18	54	3.58
Left SPL	-16	-56	66	3.86
Left EBA (rostral subdivision)	-46	-66	10	3.85
Left cMTG (superior division)	-40	-64	8	3.59
Left MT+	-44	-78	0	4.11
Left Visual cortex V2	-16	-100	8	3.91
Left LING	-2	-82	-6	4.03
Left FUS/FG	-22	-84	-12	4.44
Right SPL	20	-66	58	3.60
Right EBA	50	-62	-2	3.85
Right MT+	46	-70	2	3.84
Right Visual cortex V2	14	-92	20	4.03
Right LING	36	-72	-16	4.03
Right FUS/FG	0	-80	-6	3.86

*D. Watching 2nd **same intransitive** gestures vs. rest*

(Z > 2.3, p = 0.05 cluster corrected)

Region	MNI Coordinates			Peak value
	x	*y*	*z*	z-max
Left MFG	-42	20	44	2.83
Left Supramarginal Gyrus (SMG)	-56	-48	32	3.04
Left Frontal Pole	-42	40	-10	2.78
Left EBA	-46	-74	6	3.6
Left MT+	-42	-76	4	3.96
Left SFG	-2	32	48	2.95
Right SPL	32	-46	60	3.21
Right EBA	52	-66	6	3.4
Right MFG	44	30	36	2.99
Right LING	4	-78	-2	3.85
Right MT+	44	-76	-2	2.86
Right SFG	4	30	50	3.08

*E. Watching repeated **transitive** gestures (1st vs. 2nd same)*

(Z > 2.3, p = 0.05 cluster corrected)

Region	MNI Coordinates			Peak value
	x	*y*	*z*	z-max
Left MT+	-46	-70	6	3.55
Left EBA (rostral subdivision)	-48	-70	8	3.83
Left cMTG (superior division)	-52	-66	4	2.6
Left LO (inferior division)	-48	-70	8	3.94
Left LING	-2	-78	-8	2.68
Left FUS/FG	-26	-74	-10	3.39
Right MT+	38	-76	-12	3.42
Right EBA	54	-70	8	3.9

Right LING	8	-78	-6	3.59
Right FUS/FG	24	-80	-12	3.64

*F. Watching repeated **intransitive** gestures (1st vs. 2nd same)*

(Z > 2.3, p = 0.05 cluster corrected)

Region	MNI Coordinates			Peak value
	x	y	z	z-max
Left MT+	-44	-78	0	4.24
Left EBA (rostral subdivision)	-50	-72	10	4.33
Left cMTG (superior division)	-56	-64	10	3.44
Left Paracingualte Gyrus	-2	46	-2	3.57
Left LING	-2	-82	-8	2.93
Left FUS/FG	-14	-86	-16	3.54
Right MT+	28	-78	-10	3.63
Right EBA	48	-64	6	4.31
Right LO (inferior division)	48	-72	-8	4.2
Right Paracingulate Gyrus	2	54	-6	3.71
Right LING	4	-84	-8	4.09
Right FUS/FG	20	-82	-14	3.77

*G. Repetition suppression effect specific for **intransitive** gestures (vs. transitive)*

(Z > 2.3, p = 0.05 cluster corrected)

Region	MNI Coordinates			Peak value
	x	y	z	z-max
Left EBA (rostral subdivision)	-48	-64	14	3.22
Left cMTG (superior division)	-52	-64	4	3.15
Left Precuneus (anterodorsal division)	-4	-58	56	3.21
Left Precuneus (ventrocaudal division)	-4	-70	26	3.09
Right EBA	40	-56	12	3.11

*H. Watching **all transitive** gestures (vs. intransitive)*

(Z > 2.3, p = 0.05 cluster corrected)

Region	MNI Coordinates			Peak value
	x	y	z	z-max
Left EBA (anterior part)	-44	-60	14	3.54
Left cMTG (superior part)	-50	-58	-4	3.46
Left Precuneus (anterodorsal division)	-10	-62	58	3.21
Left Precuneus (ventrocaudal division)	-8	-74	30	3

GESTURE IMITATION: MAJOR CONTRASTS FROM THE MAIN EXPERIMENT

*I. Imitation of **transitive** gestures vs. rest*

(Z > 2.3, p = 0.05 cluster corrected)

Region	MNI Coordinates			Peak value
	x	y	z	z-max
Left Postcentral Gyrus	-38	-32	60	4.67
Left Supplementary Motor Area (SMA)	-4	-10	60	5.30

Left Cingulate Gyrus (anterior division)	-2	6	40	4.34
Left Central Opercular Cortex	-46	4	4	4.21
Left SPL	-32	-46	62	4.35
Left Thalamus	-14	-18	10	4.42
Left Putamen	-24	-2	4	4.60
Left Cerebellum	-32	-56	-26	3.84
Right Precentral Gyrus	58	8	12	3.70
Right SMA	4	-8	58	4.17
Right Cingulate Gyrus (anterior division)	2	10	36	4.32
Right Thalamus	12	-4	12	3.06
Right Putamen	24	0	6	4.00
Right Cerebellum	30	-46	-28	4.40

J. Imitation of ***intransitive*** *gestures vs. rest*

(Z > 2.3, p = 0.05 cluster corrected)

Region	MNI Coordinates			Peak value
	x	*y*	*z*	z-max
Left Precentral Gyrus	-42	-12	62	4.65
Left Postcentral Gyrus	-34	-28	70	5.30
Left SMA	-4	-10	62	4.65
Left Thalamus	-12	-18	10	3.59
Left Putamen	-26	-2	4	4.01
Right Precentral Gyrus	60	10	16	3.45
Right SMA	2	-8	50	4.30
Right Cingulate Gyrus (anterior division)	4	12	38	4.03
Right Putamen	26	0	8	3.75

K. Imitation of ***transitive*** *gestures* ***vs. intransitive***

(Z > 2.3, p = 0.05 cluster corrected)

Region	MNI Coordinates			Peak value
	x	*y*	*z*	z-max
Left Superior Frontal Gyrus (SFG)	-14	-6	70	3.46
Left Precentral Gyrus	-26	-10	54	3.66
Left Postcentral Gyrus	-28	-36	66	3.14
Left SPL	-26	-48	68	2.88
Left SMA	-2	-4	58	3.05

L. Imitation of ***intransitive*** *gestures* ***vs. transitive***

(Z > 2.3, p = 0.05 cluster corrected)

Region	MNI Coordinates			Peak value
	x	*y*	*z*	z-max
Left Paracingulate Gyrus	-6	52	12	3.20
Right Paracingulate Gyrus	4	50	16	3.08
Right Central Opercular Cortex	46	-4	12	3.19

LOCALIZER SCANS: MAJOR CONTRASTS				
M. ***EBA*** *localizer (bodies (–scrambled bodies) vs. furniture (–scrambled furniture*)				
(Z > 3.1, p = 0.05 cluster corrected)				

Region	MNI Coordinates			Peak value
	x	*y*	*z*	z-max
Left LO superior division (EBA)	-48	-70	12	5.72
Left Cingulate Gyrus	-2	40	12	4.30
Left Paracingulate Gyrus	-2	44	20	4.41
Left SFG	-10	22	64	4.12
Right LO superior division (EBA)	50	-72	8	5.46
Right Paracingulate Gyrus	2	44	26	4.43
Right Frontal Pole	14	54	34	4.57

N. ***MT+*** *localizer (moving vs. stationary patterns)*

(Z > 5.4, p =0.001 voxel-corrected)

Region	MNI Coordinates			Peak value
	x	*y*	*z*	z-max
Left LO inferior division (hMT+)	-46	-78	2	7.12
Right LO inferior division (hMT+)	48	-70	2	5.72

Average peak coordinates (in MNI space), and their peak values (Z statistics) in all major functional areas and regions identified in this study are listed.

Supplemental ROI analyses

In the subdivision that belongs to the rostral EBA (*rEBA*; see **Supplemental Figure 1A**, upper inset on the right), a 2 (gesture: transitive, intransitive) by 3 (context: first gesture, second same, second different) repeated-measures ANOVA revealed a significant main effect of *gesture* ($F_{(1,11)} = 14.332$; $p < 0.05$; ${}_{p}\eta^2 = 0.56$; alpha = 0.93), such that the signal associated with watching intransitive gestures was significantly weaker than for transitive gestures. There was also a trend towards a main effect of *context* ($F_{(2,22)} = 2.782$; $p = 0.084$; ${}_{p}\eta^2 = 0.202$), but signal decreases across the two categories for watching the repeated gestures were not substantial enough. The *gesture* by *context* interaction was not significant, either ($F_{(2,22)} = 1.457$; $p = 0.254$). Yet, both the whole brain (voxelwise) analysis and the inspection of the the

upper inset **in Fig. 1A** clearly indicates that rEBA reveals different adaptation for the two gesture categories. Indeed, as shown by the *a priori* t-test, the signal decreases for the same back-to-back intransitive and transitive gestures were significantly different ($t_{(11)} = -2.751$; $p < 0.05$). In the subdivision that belongs to the caudal MTG *(cMTG ROI;* see the lower inset of **Supplemental Figure 1A**), a repeated-measures ANOVA also showed a significant main effect of *gesture* ($F_{(1,11)} = 8.324$; $p < 0.05$; $_p\eta^2 = 0.431$; alpha = 0.748) and again the signal associated with watching intransitive gestures was weaker than for transitive pantomimes. A main effect of *context* ($F_{(2,22)} = 2.503$; $p = 0.105$) and an interaction were not significant ($F_{(2,22)} = 2.108$; $p = 0.145$). As before, such an ANOVA was not particularly informative because a mere inspection of the graph (the lower inset on the right) indicates that the signals associated with watching the first gestures from both categories did not differ. This is indeed the case ($t_{(11)} = 1.54$; $p = 0.150$) Even more importantly, and consistent with the whole brain analysis, the *a priori* t-test also revealed that the decreases of signal for the same back-to-back intransitive gestures were significantly greater ($t_{(11)} = -2.473$; $p < 0.05$).

Supplemental Figure 1B, shows all the major subdivisions of the occipito-temporal cortex.

In the left *anterodorsal Precuneus ROI* (see **Supplemental Figure 1C**, upper inset on the left), a repeated-measures ANOVA again revealed a main effect of *gesture* ($F_{(1,11)} = 6.789$; $p < 0.05$; $_p\eta^2 = 0.382$; alpha = 0.661) in which, consistent with the voxelwise analysis, the signal associated with watching transitive gestures was significantly higher. A main effect *context* was also significant ($F_{(2,22)} = 23.814$; $p < 0.001$; ; $_p\eta^2 = 0.684$; alpha = 1), wherein gesture repetition resulted in substantial signal increases regardless of whether the meaning of the gesture was the same or different (Bonferroni corrected $p < 0.05$ for both comparisons).

Finally, there was a clear trend towards an interaction ($F_{(2,22)} = 3.155$; $p = 0.062$; $_p\eta^2 = 0.684$), but signal increase associated with the repeated transitive gesture was not strong enough. Notably, although the voxels belonging to this ROI formed a subset of a larger cluster (spanning the superior SPL and the anterior precuneus) revealed by a direct contrast searching for adaptation (here: signal decrease or suppression) for repeated intransitive (vs. transitive) gestures, a glimpse at the % signal change graph clearly indicates that no suppression was present there. In fact, for both gesture categories there were rather clear signal increases (repetition enhancement). Yet, as shown by the *a priori* t-test ($t_{(11)} = -2.583$; $p < 0.05$) the signal increase for repeated transitive gestures was in fact significantly stronger.

In the left *ventrocaudal Precuneus* ROI (see **Supplemental Figure 1C**, lower inset on the left), a subset of a cluster involving also the parieto-occipital cortex, a repeated-measures ANOVA showed a significant main effect of *gesture* ($F_{(1,11)} = 10.355$; $p < 0.05$; $_p\eta^2 = 0.485$; alpha = 0.833) with higher activity associated with watching transitive pantomimes. Neither a main effect of *context* ($F_{(2,22)} = 1.281$; $p = 0.298$) nor an interaction was significant ($F_{(2,22)} = 1.550$; $p = 0.235$). As for adPreCun, an *a priori* t-test ($t_{(11)} = - 3.758$; $p < 0.01$) revealed a significantly greater signal increase for transitive gestures during watching videos with the same meaning. Again, this effect is consistent with greater repetition enhancement effect for this gesture category.

Supplemental Figure 1

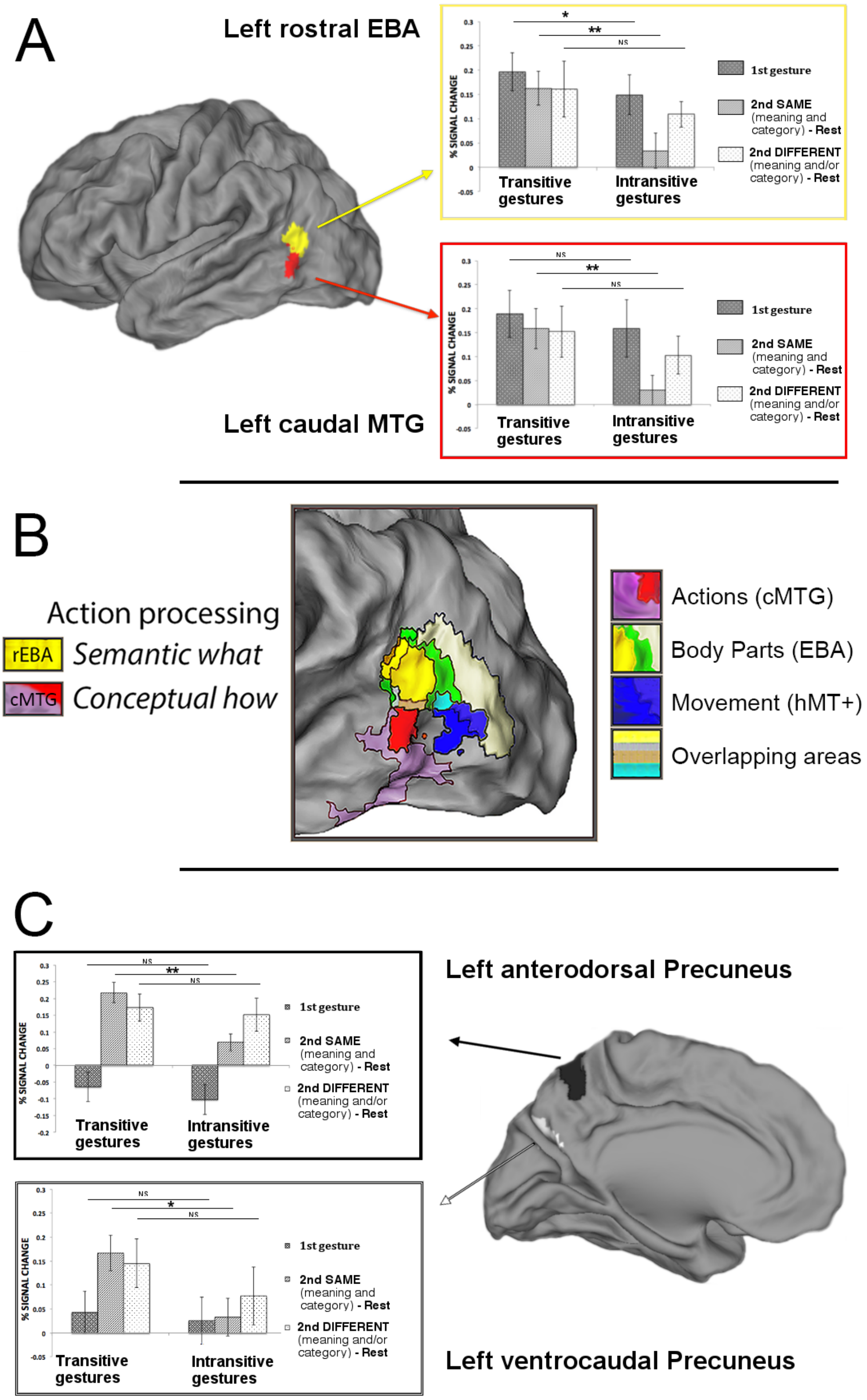

Supplemental Figure 1. Additional region of interest (ROI) analyses. *(A) Repetition suppression in the rostral extrastriate body area (rEBA) and the caudal middle temporal*

gyrus (cMTG). As shown by our localizer runs and previous studies, this occipito-temporal cluster belongs to two functional areas: its superior division to rEBA, and the inferior one to cMTG. In both cases, significant repetition suppression was exclusive to the same intransitive gestures. The critical difference between the rEBA and cMTG pattern of RS was their baseline activities, which did not differ for cMTG. *(B) All the occipito-temporal functional subdivisions from our localizer runs*. The putative functions of these areas are described and color coded. The parcels from previous studies[1,2] were also used. *(C). Repetition enhancement in the ventrocaudal precuneus (vcPreCun), and anterodorsal precuneus (adPreCun)*. The most pronounced and significant increases of activity following gesture repetition were found in both of the ROIs for transitive gestures. Asterisks indicate all the significant differences with the Bonferroni-corrected P values of at least 0.05 (*) or 0.01 (**); 'NS' indicates substantial but not significant differences.

Supplemental Figure 2

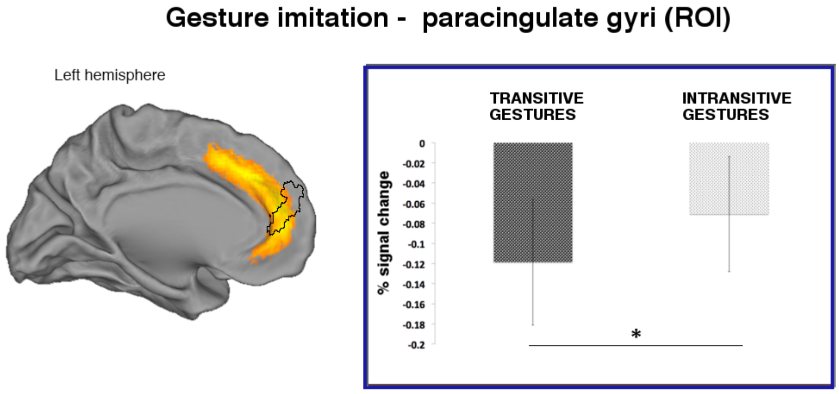

Supplemental Figure 2. Signal modulations observed during gesture imitation. Imitation of intransitive gestures as compared to imitation of transitive gestures (tool use pantomimes) resulted in significantly weaker signal inhibition within the bilateral paracingulate gyrus ($t_{(11)} = -2.251$; $p < 0.05$). Only the medial surface of the left hemisphere is presented here. The actual cluster of significant difference which was revealed in a direct contrast (i.e., intransitive vs. transitive imitation) in the whole brain analysis is shown in a schematic form. The independent bilateral paracingulate gyrus ROI was obtained from the *Harvard-Oxford atlas* implemented in the FSL package. The probabilistic map was thresholded at 30% of its lower probability tail.

References:

1. Kroliczak, G. & Frey, S. H. A common network in the left cerebral hemisphere represents planning of tool use pantomimes and familiar intransitive gestures at the hand-independent level. *Cereb Cortex* **19**, 2396-2410, doi:10.1093/cercor/bhn261 (2009).
2. Julian, J. B., Fedorenko, E., Webster, J. & Kanwisher, N. An algorithmic method for functionally defining regions of interest in the ventral visual pathway. *Neuroimage* **60**, 2357-2364, doi:10.1016/j.neuroimage.2012.02.055 (2012).
